# Implementation of Universal School Meals during COVID-19 and beyond: Challenges and Benefits for School Meals Programs in Maine

**DOI:** 10.3390/nu14194031

**Published:** 2022-09-28

**Authors:** Juliana F. W. Cohen, Michele Polacsek, Christina E. Hecht, Ken Hecht, Margaret Read, Deborah A. Olarte, Anisha I. Patel, Marlene B. Schwartz, Lindsey Turner, Monica Zuercher, Wendi Gosliner, Lorrene D. Ritchie

**Affiliations:** 1 Center for Health Inclusion, Research and Practice (CHIRP), Merrimack College, 315 Turnpike Street, North Andover, MA 01845, USA; 2 Department of Public Health and Nutrition, Merrimack College, 315 Turnpike Street, North Andover, MA 01845, USA; 3 Department of Nutrition, Harvard T.H. Chan School of Public Health, 677 Huntington Ave, Boston, MA 02115, USA; 4 Center for Excellence in Public Health, University of New England, 716 Stevens Ave, Portland, ME 04103, USA; 5 Nutrition Policy Institute, Division of Agriculture and Natural Resources, University of California, Oakland, CA 94607, USA; 6 Share Our Strength, No Kid Hungry, Washington, DC 20005, USA; 7 Division of General Pediatrics, Stanford University, Palo Alto, CA 94304, USA; 8 Rudd Center for Food Policy and Obesity, Department of Human Development and Family Sciences, University of Connecticut, 1 Constitution Plaza, Suite 600, Hartford, CT 06103, USA; 9 College of Education, Boise State University, 1910 University Drive, Boise, ID 83725, USA

**Keywords:** universal school meals, nutrition, community eligibility provision, breakfast, lunch

## Abstract

School meals play a major role in supporting children’s diets and food security, and policies for universal school meals (USM) have the potential to contribute to positive child health outcomes. During the COVID-19 pandemic, schools provided free school meals to all students in the United States, but this national USM policy ended in school year (SY) 2022–2023; however, a few states have adopted policies to continue USM statewide for SY 2022–2023. Research examining the challenges and strategies for successful continuation of USM is essential, along with studying pandemic-related challenges that are likely to persist in schools. Therefore, we conducted a study in Maine (with a USM policy) to evaluate the impact of COVID-19 and the concurrent implementation of USM, as well as examine differences in implementation by school characteristics, throughout the state. A total of *n* = 43 school food authorities (SFAs) throughout Maine completed surveys. SFAs reported multiple benefits of USM including increased school meal participation; reductions in the perceived stigma for students from lower-income households and their families; and no longer experiencing unpaid meal charges and debt. SFAs also experienced challenges due to the COVID-19 pandemic, particularly regarding costs. When considering future challenges, most respondents were concerned with obtaining income information from families, product and ingredient availability, and the costs/financial sustainability of the school meal programs. Overall, USM may have multiple important benefits for students and schools, and other states should consider implementation of a USM policy.

## 1. Introduction

In the United States, the federal school meal programs play a major role in supporting children’s nutrition, food security, and overall health [1]. Research suggests that many children have their most nutritious meals of the day at school [2,3,4], and school meals are healthier on average than meals brought from home [1,5,6,7,8]. School meals also provide an opportunity to establish longer-term healthy dietary patterns, as children’s eating preferences often persist into adulthood [2,9,10,11,12]. Nearly all US schools participate in the federal school meal programs and most use a three tiered payment system: students from households with incomes below 130% of the federal poverty level are eligible to receive free meals; those from households with incomes between 130% and 185% pay a reduced-price; and those from households above 185% pay full price [1]. However, this means-tested approach excludes many resource-constrained families at risk of food insecurity who are near eligible for free or reduced-price meals but do not qualify [13,14]. Further, some students who are eligible for free or reduced-price meals do not participate due to stigma. Students may feel shame about receiving a free school meal or their parents/caregivers may be reluctant to complete the necessary paperwork because it denotes that they are from a low-income household [15,16]. Finally, school food service departments spend time and resources tracking students’ school meal eligibility status, dedicating limited resources to program administration rather than their core job of feeding students. Therefore, universal school meal (USM) policies—where meals are available to all students in a school at no cost—have gained attention as a policy change that can achieve many goals. USM can reduce stigma; improve children’s diet-quality; nourish students for the academic demands of the school day; and benefit schools by potentially lowering administrative costs and eliminating school meal debt from households with insufficient funds to pay for meals [1,5,6,7,8,17,18,19,20,21,22].

Prior to the COVID-19 pandemic, there were several provisions supported by the United States Department of Agriculture (USDA) that enabled schools in high-poverty communities to provide free school breakfasts and/or lunches to all students: Provision 1, Provision 2, Provision 3, and Community Eligibility Provision (CEP) [17,23]. The most commonly implemented of those provisions is CEP, which was introduced as part of the Healthy Hunger-Free Kids Act of 2010 and became available nationwide to eligible schools in 2014 [24,25]. For schools or school districts to be eligible to participate in CEP, at least 40% of students must be from low-income households, which can be verified using existing administrative data (e.g., participation in other federal programs such as the Supplemental Nutrition Assistance Program). However, a majority of schools in the U.S. have not been eligible to participate in CEP, and even among eligible schools, nearly a third have opted not to participate [26].

During the COVID-19 pandemic, there was an alarming rise in the prevalence of poverty and food insecurity among households with children, disproportionately impacting communities of color [27]. In response, Congress authorized the USDA to provide waivers for all schools nationally to operate with USM from March 2020 through June 2022 (School Years [SY] 2019–2020 to 2021–2022) [28]. This national policy ended in SY 2022–2023; however, five states—California, Maine, Nevada, Vermont, and Massachusetts—have adopted policies to continue USM for all schools statewide for the 2022–2023 school year. Research examining the challenges and strategies for successful continuation of USM in these states is essential, and the findings can also inform other state and national efforts. Additionally, pandemic-related challenges that schools are facing now are likely to persist and are important to document to inform resources and policies to support schools. Therefore, we conducted a study in Maine, which is one of the states that passed legislation to continue USM indefinitely. Our aim is to evaluate the impact of COVID-19 and the concurrent implementation of USM, as well as examine differences in implementation by school characteristics, throughout the state.

## 2. Materials and Methods

### 2.1. Participants

During the spring of 2022, all K-12 public and charter school food authorities (SFA) in Maine (*n* = 121) were sent a recruitment email by Full Plates Full Potential, a local anti-hunger organization. The recruitment email described the study and provided a link to an online survey. Recruitment procedures yielded a 36% participation rate and respondents were provided with a USD25 incentive for participation.

### 2.2. Measures and Data Collection Procedures

The race/ethnicity of the student populations for the participating SFAs were obtained using data from the National Center for Education Statistics (NCES). The urbanicity of each SFA was determined using Rural–Urban Commuting Area (RUCA) Codes from the US Department of Agriculture’s Economic Research Service. RUCA Codes categorize US census tracts using measures of population density, urbanization, and commuting. SFAs were categorized as: (1) urban (RUCA code 1); (2) suburban (RUCA codes 2–3); (3) large rural (RUCA codes 4–6); and (4) rural/small town (RUCA codes 7–10).

The online survey was a 58-item questionnaire administered electronically using Qualtrics (Version March, 2022, Provo, UT, USA). Reminder emails were sent two weeks after the initial email and the survey was open for up to four weeks. The survey included selected questions from the USDA’s “SFA Survey on Supply Chain Disruption” surveys; the School Nutrition Meal Cost Study [29,30,31,32,33,34,35,36,37]; and questions developed by the research team in collaboration with nutrition/school policy advocacy organizations (i.e., School Nutrition Association, Center for Science in the Public Interest, and the Urban School Food Alliance) and anti-hunger organizations (i.e., Full Plates Full Potential, and Share our Strength). This survey was also designed with academic partners in California and the California Department of Education who were developing a complementary statewide survey. The survey questions were pilot tested with school nutrition professionals (e.g., food service directors and cafeteria managers) and updated based on feedback. The final survey included questions regarding the COVID-19 pandemic and USM during the school year in which data were collected (SY 2021–2022); perceived barriers for students to participate in school breakfast and lunch; and concerns and resources needed for continued implementation of USM when Maine’s policy goes into effect in SY 2022–2023. The present analysis focuses specifically on survey questions regarding (1) challenges related to COVID-19 and/or supply-chain issues; (2) changes due to implementing USM; and (3) concerns and resources needed to continue Maine’s implementation of USM during the next school year. Survey questions regarding challenges regarding COVID-19 used a 4-point scoring system that ranged from “1 = not a challenge” to “4 = significant challenge”. Changes due to USM used a 5-point scoring system that ranged from “1 = decreased greatly” to “5 = increased greatly”. Concerns used a 4-point scoring system that ranged from “1 = not a concern” to “4 = serious concern”. Finally, resources needed for next school year used a 4-point scoring system that ranged from “1 = not needed” to “4 = needed a lot”. The Institutional Review Board at Merrimack College approved this study (Project identification code IRB-FY21-22-19).

### 2.3. Statistical Analyses

Analysis of Variance (ANOVA) was used to examine the differences in survey responses regarding the impact of COVID-19 and USM (current and anticipated for next year) by school demographics, including urbanicity (coded as an ordinal variable) and prior CEP/Provision 2/3 status (hereafter referred to as CEP). Average scores by urbanicity and prior CEP status were calculated using least squares means regression. Other school demographic characteristics (e.g., race/ethnicity [based on average student population], SFA food preparation methods [central kitchen or prepared on site], and participation in food cooperatives) were examined but were not statistically significant and therefore were not included in the final models. Survey questions regarding the perceived impact of USM during the school year (SY 2021–2022) were only asked among schools that had not previously participated in CEP (i.e., had not previously provided USM) and therefore final models only examined differences by urbanicity. All analyses were conducted using SAS (Version 9.4. SAS Institute Inc., Cary, NC, USA).

## 3. Results

Fifty-three survey responses were received. From those, responses that were either empty or only had personal information of the respondent without responses to any of the additional survey questions (*n* = 7) and duplicate responses were removed (*n* = 3) with the most complete survey used. Therefore, *n* = 43 SFAs were represented by survey respondents (36% response rate). The majority of respondents (72%) were school nutrition directors or foodservice directors, while 14% were supervisors or managers, and 14% played another role within the district (Table 1). Roughly a quarter of respondents (26%) had been in that role within the SFA between 1–4 years, over a third (35%) had that role for 5–9 years, and over a third (37%) had been in that role for ten or more years. Only one respondent reported being in their role for less than one year. The majority (63%) had educational attainment less than an undergraduate degree, with 37% having a college degree or advanced training. Roughly half (54%) of SFAs were in small town/rural areas, with the remaining evenly distributed among large rural, suburban, and urban areas. The percentage of student populations served that were white averaged 93%. Over half (56%) of SFAs prepared school meals on site, 35% utilized district nutrition services or a central kitchen to prepare foods, and 9% used a combination of both. A majority (70%) reported that they participated in a food-purchasing cooperative. The majority of schools (83%) implemented USM for the first time during the pandemic; only 17% had participated in CEP at a district-wide level prior to the national USM policy. The most common USM policy was providing free school breakfasts, with roughly a third of the schools providing this prior to the national policy.

### 3.1. Challenges Related to COVID-19 and/or Supply-Chain Issues during the School Year (SY) 2021–2022

**Figure 1:**
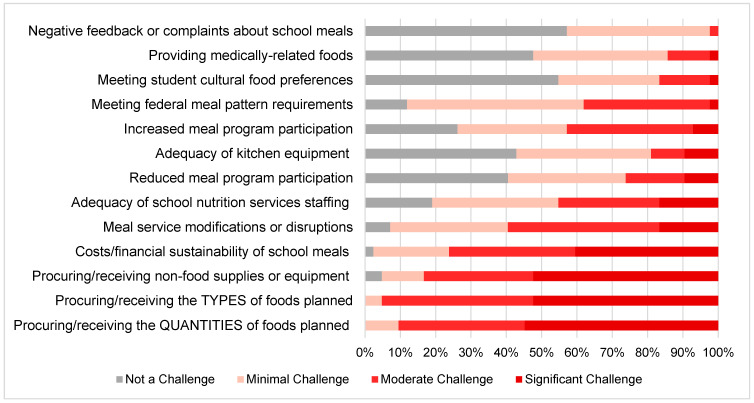
Perceived Challenges Related to the COVID-19 Pandemic among School Food Authorities in Maine (*n* = 43).

When examining challenges related to COVID-19 and/or supply chain issues, over 75% of responding SFAs reported moderate or significant challenges related to procurement (the quantities and types of food, as well as non-food supplies/equipment) and the costs/financial sustainability of school meals (Figure 1). With respect to procurement, over half of respondents reported significant challenges related to procuring or receiving the quantities of foods or beverages ordered (55%), the types of foods/beverages ordered (52%), and non-food supplies or equipment (52%). With respect to the impact of the pandemic on school finances, 42% of respondents reported that the current reimbursement rate was not sufficient to cover the cost of breakfast, and 51% reported that it was not sufficient to cover the cost of lunch (Supplemental Figure S1). Among respondents that reported the current reimbursement rate was not sufficient for breakfast, 18% reported that reimbursement covered ≤50% of the full cost of producing meals, 41% reported that it covered 51–75%, and 41% responded that it covered ≥75% of the cost. Among those that reported lunch reimbursement was not sufficient, 43% reported that USDA reimbursement covered ≤50% of the full production cost, 19% reported that it covered 51–75%, and 38% responded that it covered ≥75% of the full cost of lunch. The most frequently cited causes of school meal deficits were the cost of food (reported by 100% of SFAs experiencing a deficit), school nutrition services labor costs (90% of SFAs), and supply costs (71% of SFAs). Among all SFAs, the average amount reported for meal reimbursement to be sufficient to cover the full cost of producing meals to meet all federal nutrition standards, and also appeal to students was USD2.75 for breakfast (USD0.33 more than the concurrent USDA SY 2021–2022 reimbursement rate of USD2.42 for breakfast) and USD4.77 for lunch (USD0.52 more than the reimbursement rate of USD4.25 for lunch) [38]. Conversely, the majority of respondents reported either minimal or no negative feedback/complaints regarding school meals from parents or students, or challenges meeting students’ cultural food preferences or the needs of children with medically related food requirements.

When examining differences by urbanicity, challenges related to the cost/financial sustainability of the school meal program were inversely associated with urbanicity, with greater challenges reported among rural SFAs (β = 0.26; *p* = 0.01 (Table 2)); respondents in rural SFAs had average survey scores of 3.4 (significant/moderate challenge) compared to 2.7 (moderate/minimal challenge) in urban schools. Conversely, respondents in urban SFAs reported greater challenges associated with meeting students’ cultural preferences (β = 0.21; *p* = 0.04). Respondents from SFAs that did not previously participate in CEP reported that procuring and receiving a sufficient amount of non-food supplies was more challenging compared to those previously participating in CEP (average score of 3.4 vs. 2.7; *p* = 0.04). Respondents from CEP schools were more likely to report negative feedback or complaints about the school meals from parents or students (1.9 vs. 1.4; *p* = 0.02), although this was a minimal challenge overall. There were no other significant differences among the perceived challenges associated with the pandemic by urbanicity or prior CEP status.

### 3.2. Perceptions of Changes Due to Implementing USM during the School Year (SY) 2021–2022

**Figure 2:**
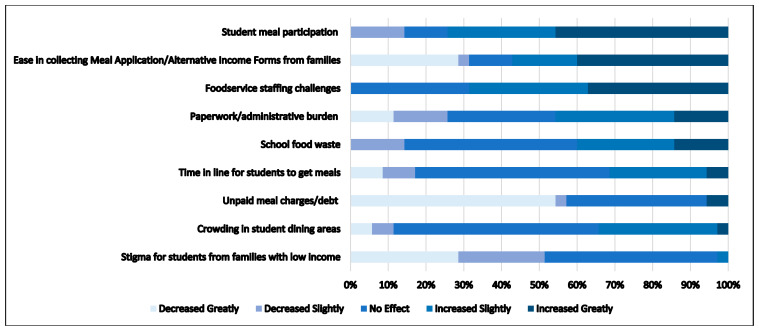
Perceptions of Changes Due to Implementing Universal School Meals in School Year 2021–2022 among School Food Authorities in Maine (*n* = 35). Surveys restricted to School Food Authorities not previously participating in the Community Eligibility Provision (CEP).

Figure 2 illustrates the reported benefits and challenges of implementing USM among the 35 SFAs that had not previously implemented USM through CEP. The greatest perceived benefit was an increase in school meal participation, with roughly three quarters of respondents (75%) reporting an increase. Similarly, over half of respondents (57%) reported greater ease regarding the collection of meal applications/alternative income forms from families. Another reported benefit was a reduction in the perceived stigma for students from lower-income households and their families, with roughly half of respondents (51%) noting that stigma decreased with the USM policy. Over half of respondents (57%) also reported that USM was associated with a decrease in the problem of unpaid meal charges and debt. Conversely, roughly two-thirds of respondents (69%) noted that the USM policy was associated with increases in foodservice staffing challenges. Results were mixed when examining crowding in student dining areas, the time that students spent in line to get meals, and perceived school food waste, with roughly half of respondents reporting that a USM policy had no effect on these cafeteria-related issues. Responses were also mixed regarding the impact on paperwork and the administrative burden, with responses nearly evenly distributed among all response categories. When examining the perceptions of USM by urbanicity, there were no significant differences for any of the responses.

### 3.3. Anticipated Concerns and Resources Needed in the Future (SY 2022–2023)

**Figure 3:**
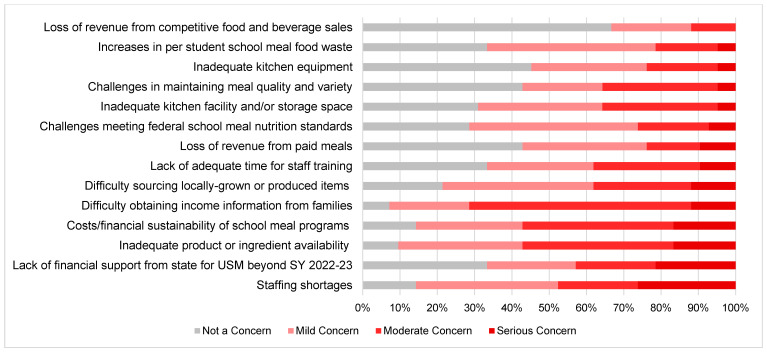
Anticipated Future Challenges among School Food Authorities in Maine (*n* = 43). Note: USM: Universal School Meals.

Several anticipated challenges (Figure 3) were reported. Focusing on the upcoming school year (SY 2022–2023), 71% of respondents were moderately or seriously concerned about obtaining income information from families, which schools use to obtain state and federal education funding (such as Title 1 funding). There were also continued concerns about inadequate product/ingredient availability and the costs/financial sustainability of the school meal programs, with over half of SFAs (57%) reporting moderate to serious concerns for both issues. Other commonly reported concerns included staffing shortages (48%); lack of financial support from Maine for USM beyond next school year (43%); difficulty obtaining locally grown or produced items (38%); insufficient time for staff training (38%); inadequate kitchen facility and storage space (36%); and challenges maintaining meal quality and variety (36%). However, two-thirds of respondents were not concerned about revenue losses from competitive food and beverage sales, only 20% were moderately or seriously concerned about increases in school meal food waste, and only about 25% were moderately or seriously concerned about meeting the federal school meal nutrition standards.

Table 3 provides the differences in anticipated challenges among SFAs by urbanicity and CEP status. When examining differences by urbanicity, respondents from rural SFAs reported greater concerns regarding the ability to maintain meal quality and variety compared with those from more urban SFAs (β = 0.25; *p* = 0.04); rural respondents had an average survey score of 2.4 (mild/moderate concern) compared to urban respondents with an average score of 1.6 [not a concern/mild concern]). Similarly, respondents from rural SFAs reported greater concerns in their ability to source locally grown or produced items (β = 0.34; *p* = 0.002 [average score of 1.6 for urban SFAs and 2.5 for rural schools]). Conversely, concerns related to the lack of financial support from the state for USM beyond SY 2022–2024 were lower among rural SFAs (β = −0.31; *p* = 0.03), with average survey scores of 2.0 (mild concern) among rural respondents compared to 2.6 (mild/moderate concerns) among urban ones. Concerns regarding the difficulty in obtaining income information from families were also lower in rural SFAs compared to more urban SFAs (β = −0.20; *p* = 0.04); respondents in urban SFAs had average scores of 3.1 (moderate/serious concerns) compared to 2.6 (mild/moderate concerns) in rural SFAs. There were no other significant differences among the perceived concerns by urbanicity nor were there significant differences in concerns by prior CEP status.

Three-quarters (75%) of respondents reported needing resources to help increase school meal participation. Other top needs included additional support for facilities and/or equipment (73%) and additional resources for communications and marketing to students and parents (71%). Two-thirds (66%) reported that more resources were needed to make school meals more appealing to students, and half of SFAs responded that they would benefit from resources for financial management. However, the majority of SFAs reported not needing assistance with food safety; menu planning/meal counting/claiming; cultural diversity in meal planning; or meeting special dietary needs

## 4. Discussion

This study found that SFAs in Maine experienced multiple important benefits from the USM policy implemented during the pandemic. In particular, SFAs reported markedly increased school meal participation; greater ease in collecting meal applications/alternative income forms from families; reductions in the perceived stigma for students from lower-income households and their families; and no longer experiencing unpaid meal charges and debt. At the same time, SFAs also experienced many challenges due to the COVID-19 pandemic, especially regarding costs. This concern was heightened among those working in rural districts and those that had not previously participated in CEP. When considering future challenges, most respondents were concerned with obtaining income information from families (which will continue to be required for federal school meal reimbursements as well as educational funding), product and ingredient availability, and the costs/financial sustainability of the school meal programs overall. Additionally, respondents expressed major concerns about financial support for USM from the state beyond the SY 2022–2023, especially among those from urban school districts.

The challenges faced by SFAs in this study are similar to those previously reported by a 2021 USDA survey that found that the majority of SFAs experienced labor challenges, supply chain disruptions, and limited product availability due to COVID-19 [34]. The USDA survey also found that SFAs anticipated these challenges continuing through SY 2022–2023. The present survey found that only 58% of SFAs reported breaking even financially, which is lower than the USDA survey where 71% of SFAs reported breaking even. Recognizing the challenges faced by school food service programs, Congress passed the Keep Kids Fed Act in June 2022. This law increases funding for school meals by USD0.15 per breakfast and USD0.40 per lunch [39]. Although important, these increases are still substantially lower than the amounts SFAs in the present study reported needing, which was an average of USD0.33 more per breakfast and USD0.52 per lunch. Our findings suggest that the reimbursement rates may need to be increased even further to ensure healthy and appealing meals are provided to students. Additionally, alternative mechanisms (i.e., that do not involve school nutrition programs) are needed to measure school poverty in order to allocate education funding; several ideas were highlighted in a recent research brief [40].

This study had several limitations. First, this was a cross-sectional study conducted in only one state with a low percentage of students who were racial or ethnic minorities. More research in other states with diverse student populations is warranted. Additionally, the sample size was relatively small even though a third of the SFAs within the state were represented. This study was further strengthened by representation among SFAs in small towns/rural areas, large rural, suburban, and urban districts and the results are therefore likely generalizable to other similar states. Given the lack of data on USM policies on students and household finances, future studies should examine the impact of USM on students and households, such as on food security.

## 5. Conclusions

This is one of the first studies to examine the impact of pandemic-related USM on non-CEP schools within the United States. Study findings suggest there are multiple important benefits of USM to schools not previously eligible to provide free school meals to all students. In particular, the reported increases in meal participation and reductions in stigma may help to promote nutrition equity among students. Schools have also faced many challenges due to the COVID-19 pandemic and anticipate that school meal program challenges will continue. Policy makers at the local, state, and federal levels should further consider how to support schools to ensure they can provide healthy meals for all students

## Figures and Tables

**Table 1:** Demographic Characteristics of the participating School Food Authorities (SFAs) in Maine (*n* = 43).

Respondent Characteristics	*n* (%)
Role	
School Nutrition Director/Foodservice Director	31 (72%)
School Nutrition Supervisor/Manager	6 (14%)
Other	6 (14%)
Years in the Role	
<1 year	1 (2%)
1–4 years	11 (26%)
5–9 years	15 (35%)
10–14 years	6 (14%)
15–19 years	4 (9%)
≥20 years	6 (14%)
Highest Education	
High School/GED	9 (21%)
Some College (no degree)	12 (28%)
Associate’s Degree	6 (14%)
Bachelor’s degree	13 (30%)
Master’s degree or more	3 (7%)
SFA Characteristics	*n* (%)
Urbanicity ^1^	
Small Town/Rural	23 (54%)
Large Rural	7 (16%)
Suburban	6 (14%)
Urban	7 (16%)
Availability of Free School Meals prior to COVID-19 ^2^	
Available in All Schools (yes)	7 (17%)
Free School Breakfast ^3^	
Elementary Schools	14 (34%)
Middle Schools	12 (34%)
High Schools	9 (29%)
Free School Lunch ^3^	
Elementary Schools	9 (22%)
Middle Schools	7 (20%)
High Schools	5 (16%)
Food Preparation Location	
District nutrition services/Central kitchen	15 (35%)
School sites	24 (56%)

GED = General Educational Development Test; ^1^ Based on Rural-Urban Commuting Area (RUCA) Codes; ^2^ Based on participation in the Community Eligibility Provision, Provision 2, or Provision 3; ^3^ Responses were among those SFAs with a corresponding grade level (some SFAs did not have elementary, middle, and/or high schools).

**Table 2:** Differences in Perceived Challenges due to COVID-19 in Maine by Urbanicity and Prior CEP status ^1^.

COVID-19 Related Challenges	Urbanicity ^2^		Prior CEP Status ^3^	
	β (SE)	*p*-Value	β (SE)	*p*-Value
Costs/financial sustainability of school meal programs	**0.26 (0.10)**	**0.01**	−0.11 (0.32)	0.7
b. Procuring or receiving the *types* of foods or beverages planned	0.01 (0.08)	0.9	0.10 (0.2)	0.7
c. Procuring or receiving the *quantities* of foods or beverages planned	0.01 (0.09)	0.9	0.12 (0.28)	0.6
d. Procuring or receiving non-food supplies or equipment needed for school meals	−0.09 (0.11)	0.4	**−0.70 (0.34)**	**0.04**
e. Meeting federal meal pattern requirements	0.09 (0.09)	0.3	0.15 (0.29)	0.6
f. Meeting student cultural food preferences	**−0.21 (0.10)**	**0.04**	0.56 (0.31)	0.07
g. Meeting meal modifications for children with medically related food and nutrition needs	−0.12 (0.10)	0.2	−0.47 (0.31)	0.1
h. Adequacy of kitchen equipment	−0.16 (0.12)	0.2	−0.43 (0.37)	0.2
i. Adequacy of school nutrition services staffing	−0.14 (0.13)	0.3	0.01 (0.40)	0.9
j. Meal service modifications or disruptions (e.g., social distancing, classroom meals, need for personal protective equipment/PPE, accommodating distance learners)	−0.12 (0.11)	0.2	0.02 (0.33)	0.9
k. Increased meal program participation	0.04 (0.12)	0.7	−0.68 (0.37)	0.06
l. Reduced meal program participation	0.05 (0.13)	0.7	0.71 (0.39)	0.07
m. Negative feedback or complaints about school meals from parents or students	−0.02 (0.07)	0.7	**0.48 (0.21)**	**0.02**

Note: Boldface indicates significance. ^1^ Perceived challenge was rated by school food personnel on a scale of 1–4 (1 = Not a Challenge; 2 = Minimal Challenge; 3 = Moderate Challenge; 4 = Significant Challenge), ^2^ Based on Rural–Urban Commuting Area (RUCA) Codes from the US Department of Agriculture’s Economic Research Service (Ordinal variable categorized as 1 = Urban; 2 = suburban, 3 = large rural; 4 = small towns/rural areas), ^3^ Reference Group is not previously participating in CEP.

**Table 3:** Differences in Concerns Regarding Anticipated Future Challenges among School Food Authorities in Maine by urbanicity and prior CEP status ^1^ (*n* = 43).

Perceived Concerns	Urbanicity ^2^		Prior CEP Status ^3^	
	β (SE)	*p*-Value	β (SE)	*p*-Value
Loss of revenue from competitive food and beverage sales	−0.05 (0.09)	0.6	−0.31 (0.27)	0.5
b. Increases in per student school meal food waste	−0.06 (0.11)	0.6	−0.24 (0.35)	0.5
c. Inadequate kitchen equipment	−0.08 (0.12)	0.5	−0.32 (0.37)	0.4
d. Challenges meeting federal school meal nutrition standards	0.06 (0.12)	0.6	−0.59 (0.36)	0.1
e. Challenges in maintaining meal quality and variety	**0.25 (0.12)**	**0.04**	0.09 (0.38)	0.8
f. Inadequate kitchen facility and/or storage space	−0.23 (0.11)	0.04	0.27 (0.35)	0.4
g. Lack of adequate time for staff training	−0.08 (0.13)	0.6	−0.01 (0.40)	0.9
h. Difficulty sourcing locally grown or produced items	**0.34 (0.11)**	**0.002**	−0.23 (0.34)	0.5
i. Lack of financial support from state for USM beyond SY 2022–2023	**−0.31 (0.14)**	**0.03**	0.03 (0.45)	0.9
j. Staffing shortages	−0.23 (0.13)	0.08	−0.003 (0.40)	0.9
k. Costs/financial sustainability of school meal programs	0.22 (0.12)	0.07	−0.25 (0.37)	0.5
l. Inadequate product or ingredient availability	0.12 (0.12)	0.3	−0.14 (0.36)	0.7
m. Difficulty obtaining income information from families	**−0.20 (0.10)**	**0.04**	0.29 (−0.60)	0.9

Note: Boldface indicates significance. USM: Universal School Meals, ^1^ Perceived concern was rated by school food personnel on a scale of 1–4 (1 = Not a Concern; 2 = Mild Concern; 3 = Moderate Concern; 4 = Serious Concern), ^2^ Based on Rural–Urban Commuting Area (RUCA) Codes from the US Department of Agriculture’s Economic Research Service (Ordinal variable categorized as 1 = Urban; 2 = suburban, 3 = large rural; 4 = small towns/rural areas), ^3^ Reference Group is not previously participating in CEP.

## Data Availability

The data presented in this study are available on request from the corresponding author. The data are not publicly available due to privacy restrictions.

## Supplementary Materials

The following supporting information can be downloaded at: https://www.mdpi.com/article/10.3390/nu14194031/s1, Figure S1: Perceptions of Sufficient School Meal Reimbursement Rates among *n* = 43 School Food Authorities in Maine.

## References

[1] Fox M.K., Gearan E., Cabili C., Dotter D., Niland K., Washburn L., Paxton N., Olsho L., LeClair L., Tran V. (2019). School Nutrition and Meal Cost Study Final Report Volume 4: Student Participation, Satisfaction, Plate Waste, and Dietary Intakes. Math. Policy Res..

[2] World Health Organization (2006). Food and Nutrition Policy for Schools: A Tool for the Development of School Nutrition Programmes in the European Region.

[3] Dooris M., Poland B., Kolbe L., De Leeuw E., McCall D.S., Wharf-Higgins J. (2007). Healthy settings. Global Perspectives on Health Promotion Effectiveness.

[4] Langford R., Bonell C.P., Jones H.E., Pouliou T., Murphy S.M., Waters E., Komro K.A., Gibbs L.F., Magnus D., Campbell R. (2014). The WHO Health Promoting School framework for improving the health and well-being of students and their academic achievement. Cochrane Database Syst. Rev..

[5] Gearan E., Fox M.K., Niland K., Dotter D., Washburn L., Connor P., Olsho L., Wommack T. (2019). School Nutrition and Meal Cost Study Final Report Volume 2: Nutritional Characteristics of School Meals. Math. Policy Res..

[6] Au L.E., Rosen N.J., Fenton K., Hecht K., Ritchie L.D. (2016). Eating school lunch is associated with higher diet quality among elementary school students. J. Acad. Nutr. Diet..

[7] Farris A.R., Misyak S., Duffey K.J., Davis G.C., Hosig K., Atzaba-Poria N., McFerren M.M., Serrano E.L. (2014). Nutritional comparison of packed and school lunches in pre-kindergarten and kindergarten children following the implementation of the 2012–2013 national school lunch program standards. J. Nutr. Educ. Behav..

[8] Au L.E., Gurzo K., Gosliner W., Webb K.L., Crawford P.B., Ritchie L.D. (2018). Eating school meals daily is associated with healthier dietary intakes: The Healthy Communities Study. J. Acad. Nutr. Diet..

[9] Mikkilä V., Räsänen L., Raitakari O., Pietinen P., Viikari J. (2005). Consistent dietary patterns identified from childhood to adulthood: The cardiovascular risk in Young Finns Study. Br. J. Nutr..

[10] Movassagh E.Z., Baxter-Jones A.D., Kontulainen S., Whiting S.J., Vatanparast H. (2017). Tracking dietary patterns over 20 years from childhood through adolescence into young adulthood: The Saskatchewan Pediatric Bone Mineral Accrual Study. Nutrients.

[11] Whitaker R.C., Wright J.A., Pepe M.S., Seidel K.D., Dietz W.H. (1997). Predicting obesity in young adulthood from childhood and parental obesity. N. Engl. J. Med..

[12] Lytle L.A., Seifert S., Greenstein J., McGovern P. (2000). How do children’s eating patterns and food choices change over time? Results from a cohort study. Am. J. Health Promot..

[13] Fleischhacker S., Campbell E. (2020). Ensuring equitable access to school meals. J. Acad. Nutr. Diet..

[14] Potamites E., Gordon A. (2010). Children’s Food Security and Intakes from School Meals. https://ageconsearch.umn.edu/record/292081/.

[15] Mirtcheva D.M., Powell L.M. (2009). Participation in the national school lunch program: Importance of school-level and neighborhood contextual factors. J. Sch. Health.

[16] Moore Q., Hulsey L., Ponza M. (2009). Factors Associated with School Meal Participation and the Relationship between Different Participation Measures. https://ageconsearch.umn.edu/record/292073/.

[17] Cohen J.F., Hecht A.A., McLoughlin G.M., Turner L., Schwartz M.B. (2021). Universal School Meals and Associations with Student Participation, Attendance, Academic Performance, Diet Quality, Food Security, and Body Mass Index: A Systematic Review. Nutrients.

[18] Gross S.M., Kelley T.L., Augustyn M., Wilson M.J., Bassarab K., Palmer A. (2019). Household Food Security Status of Families with Children Attending Schools that Participate in the Community Eligibility Provision (CEP) and Those with Children Attending Schools that are CEP-Eligible, but Not Participating. J. Hunger Environ. Nutr..

[19] Beaton M., Craig P., Katikireddi S., Jepson R., Williams A. (2014). Evaluability Assessment of Free School Meals for All Children in P1 to P3. Proj. Report. NHS Health Scotl. Edinb..

[20] Poppendieck J. (2011). Free for All: Fixing School Food in America.

[21] Wahlstrom K.L., Begalle M.S. (1999). More than test scores: Results of the Universal School Breakfast Pilot in Minnesota. Top. Clin. Nutr..

[22] Cullen K.W., Chen T.-A. (2017). The contribution of the USDA school breakfast and lunch program meals to student daily dietary intake. Prev. Med. Rep..

[23] Brown K. (2009). School Meal Programs: Experiences of the States and Districts That Eliminated Reduced-Price Fees. Report to the Chairman, Committee on Education and Labor, House of Representatives.

[24] United States Department of Agriculture (2012). National School Lunch Program and School Breakfast Program: Nutrition Standards for All Foods Sold in School as Required by the Healthy, Hunger-Free Kids Act of 2010. Fed Regist.

[25] Levin M., Neuberger Z. (2013). A guide to implementing community eligibility. Food Res. Action Cent. Cent. Budg. Policy Priorities Oct..

[26] Billings K., Carter J. (2020). Serving Free School Meals through the Community Eligibility Provision (CEP): Background and Participation. Congr. Res. Serv. May.

[27] Feeding America The Impact of the Coronavirus on National Food Insecurity in 2020 & 2021. https://www.feedingamerica.org/sites/default/files/2021-03/National%20Projections%20Brief_3.9.2021_0.pdf.

[28] United States Department of Agriculture FNS Responds to COVID-19. https://www.fns.usda.gov/coronavirus.

[29] U.S. Department of Agriculture (2019). School Nutrition and Meal Cost Study-School Meal Costs and Revenues.

[30] U.S. Department of Agriculture (2022). Results of the U.S. Department of Agriculture, Food and Nutrition Service-Administered School Food Authority Survey on Supply Chain Disruptions. https://www.fns.usda.gov/cn/results-fns-administered-school-food-authority-survey-supply-chain-disruption.

[31] U.S. Department of Agriculture (2021). USDA Distributes $1.5 Billion to Strengthen School Meal Program. Food and Nutrition Service (FNS). https://www.usda.gov/media/press-releases/2021/12/17/usda-distributes-15-billion-strengthen-school-meal-program.

[32] California Department of Education (2015). California Healthy Kids Study Key Informant Interview Guide for School Food & Nutrition Environment. https://www.cde.ca.gov/ls/he/at/chks.asp.

[33] Centers for Disease Control and Prevention (2006). School Health Policies and Programs Study 2006-Food Service District Questionnaire. https://www.cdc.gov/healthyyouth/shpps/2006/factsheets/pdf/fs_overview_shpps2006.pdf.

[34] U.S. Department of Agriculture (2021). SFA Survey on Supply Chain Disruption.

[35] Rider C., Linares A., Kao J., Becker C., Woodward-Lopez G. (2021). Assessing Healthful Eating and Physical Activity Practices in Places Children Learn. J. Ext..

[36] Ritchie L., Wakimoto P., Woodward-Lopez G., Thompson F., Loria C., Wilson D., Kao J., Crawford P., Webb K. (2015). The Healthy Communities Study Nutrition Assessments: Child Diet and the School Nutrition Environment. Am. J. Prev. Med..

[37] Hoffman J.A., Rosenfeld L., Schmidt N., Cohen J.F., Gorski M., Chaffee R., Smith L., Rimm E.B. (2015). Implementation of competitive food and beverage standards in a sample of Massachusetts schools: The NOURISH study (Nutrition Opportunities to Understand Reforms Involving Student Health). J. Acad. Nutr. Diet..

[38] U.S. Department of Agriculture (2020). Summer Food Service Program. https://www.federalregister.gov/documents/2020/12/31/2020-29093/summer-food-service-program-2021-reimbursement-rates.

[39] United States Department of Agriculture (2022). Biden Administration Takes Additional Steps to Strengthen Child Nutrition Programs.

[40] Hecht A., Dunn C., Turner L., Fleischhacker S., Kenney E., Bleich S. Improving Access to Free School Meals: Addressing Intersections between Universal Free School Meal Approaches and Educational Funding. https://healthyeatingresearch.org/wp-content/uploads/2021/07/HER-CEP-Policy-Brief.pdf.

