# Supplementary material for: Implementation of Universal School Meals during COVID-19 and beyond: Challenges and Benefits for School Meals Programs in Maine
Source: Nutrients. 2022 Sep 28;14(19):4031. doi: 10.3390/nu14194031 (PMC9571988; doi:10.3390/nu14194031)

Supplemental Figure S1. Perceptions of Sufficient School Meal Reimbursement Rates among n=43 School Food Authorities in Maine

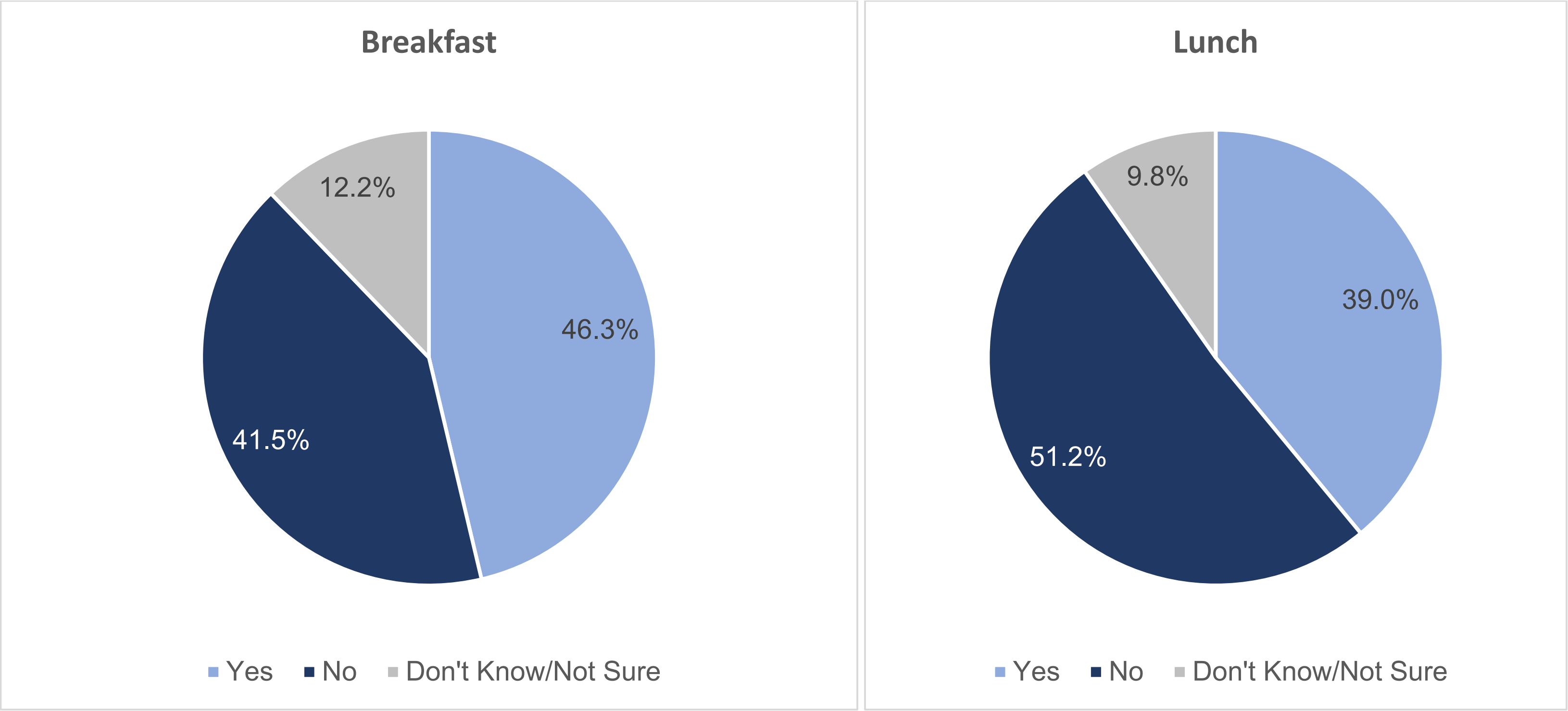

Supplement: Supplementary file 1 [file nutrients-14-04031-s001.zip › nutrients-1892872-supplementary.pdf]
